# Auditory sequence analysis and phonological skill

**DOI:** 10.1098/rspb.2012.1817

**Published:** 2012-09-05

**Authors:** Manon Grube, Sukhbinder Kumar, Freya E. Cooper, Stuart Turton, Timothy D. Griffiths

**Affiliations:** Newcastle Auditory Group, Medical School, Newcastle University, Framlington Place, Newcastle-upon-Tyne NE2 4HH, UK

**Keywords:** auditory sequence analysis, pitch, rhythm, phonological skill, adolescence, language

## Abstract

This work tests the relationship between auditory and phonological skill in a non-selected cohort of 238 school students (age 11) with the specific hypothesis that sound-sequence analysis would be more relevant to phonological skill than the analysis of basic, single sounds. Auditory processing was assessed across the domains of pitch, time and timbre; a combination of six standard tests of literacy and language ability was used to assess phonological skill. A significant correlation between general auditory and phonological skill was demonstrated, plus a significant, specific correlation between measures of phonological skill and the auditory analysis of short sequences in pitch and time. The data support a limited but significant link between auditory and phonological ability with a specific role for sound-sequence analysis, and provide a possible new focus for auditory training strategies to aid language development in early adolescence.

## 1. Introduction

Speech has a complex acoustic structure based on spectro-temporal patterns at multiple time scales ranging from tens of milliseconds at the segmental level to the suprasegmental level of phrases and sentences spanning several seconds [1–12]. This work addresses the nature of the relationship between auditory processing and phonological skill with a systematic approach.

Previous work on auditory ability and language skills and disorders mainly used basic stimuli comprising single sounds as opposed to sound sequences. Studies of typical language development for instance, have shown a correlation between pitch discrimination and reading ability at age 4–5 (*n* = 18) [13], and between frequency-modulation (FM) detection at speech-related rates (2, 40 Hz) and non-word reading at age 10 (*n* = 32) [14]. Studies of auditory processing in dyslexic compared with typically developing individuals have shown deficits in FM detection [15–17], frequency discrimination [18], amplitude rise-time discrimination [15] and discrimination of spectral changes [19,20]. Basic auditory processing deficits have also been reported in specific language impairment (SLI): frequency discrimination [21] and backward masking [22]. However, there have been failures to replicate a consistent relationship between such deficits and impaired language development or dyslexia [10,23–25].

Inconsistencies in previous studies may in part be due to the testing of small selected samples and can be addressed by studying larger unselected cohorts. A better understanding of typical language development may be needed before developmental disorders including dyslexia can be understood. An assessment of basic auditory processing of frequency, duration and modulation in over 500 typically developing undergraduates did not support a role for these skills in speech and language ability, and neither did the testing of backward-masking in 465 children at school entry [26]. A recent study of 1469 6–11-year olds assessed the relationship between verbal and non-verbal cognitive ability and basic auditory processing including frequency discrimination, backward and simultaneous masking [27]. Weak but significant correlations were reported between cognitive function and auditory performance but these were largely accounted for by an attentional rather than sensory measure. Another study of 120 typically developing subjects aged 7–45 [28] showed a significant correlation with reading skill for auditory temporal-order judgement and gap detection that explained 16 per cent of the variance (without control for intelligence). This relationship is in line with reports of auditory temporal-processing deficits in reading disability [29] and impaired temporal-order perception for pairs of sounds in reading-impaired or dyslexic children and adults [16,30]. However, the limited success of training and intervention programmes based on single-sound or paired stimuli [31–33], raises the question as to whether other levels of auditory analysis might be relevant.

A number of studies using more complex patterns have focused directly on the discrimination or identification of speech-type stimuli in quiet or in noise and demonstrated relationships with normal and impaired development, including dyslexia and SLI [29,34–37]. Studies on the auditory analysis of sound sequences that might close the gap between basic acoustic and speech stimuli are surprisingly rare. Pitch contour and rhythmic information have both been demonstrated to provide cues relevant to the parsing of the speech stream [38,39]. Two studies of pitch-sequence perception have shown correlations with reading skills in 4–5-year olds (*n* = 100) [40] and undergraduates (*n* = 30) [41]. The role for comparable rhythmic sequence analysis has not been studied directly, but difficulties with rhythmic amplitude modulation suggested that these are relevant to dyslexia [42–45].

**Figure 1.**
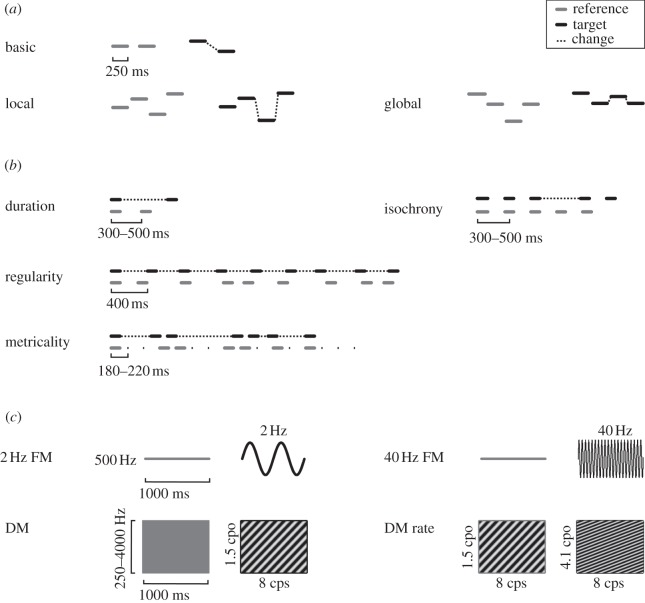
Schematic of auditory tasks. (*a*) Pitch: basic change detection (pairs of tones); local and global pitch change detection (short sequences); key violation: not shown. (*b*) Rhythm: single time-interval duration discrimination (pairs of tones); isochrony-deviation detection (short sequences); regularity detection and metrical pattern discrimination (longer sequences). (*c*) Modulation: 2 and 40 Hz FM detection; dynamic spectral modulation (DM) detection and discrimination. *x*- and *y*-axes depict time and frequency throughout, but scales are arbitrary. For each task, one reference and one target example are illustrated with their relevant features.

This study assesses the role for different levels of auditory processing in the typical development of phonological language skill in a large, non-selected cohort of early adolescents (*n* = 238; age 11). It bridges the gap between work in younger children and adults at an important point in the maturation of brain structure [46–48], the development of functions in the auditory and other domains [49–52] and the manifestation of genetic factors in language development [53]. We address the specific hypothesis that sound-sequence analysis is more relevant to phonological skill than the analysis of single sounds, using tasks in the pitch, time and timbre domains (figure 1). Phonological skill was assessed by six standard tests that required spoken or written output in response to spoken or written input using words and non-words. Correlation was sought between principal components of either dataset that might reflect general auditory and phonological mechanisms, and between the task-specific sequence-based and basic measures of auditory analysis and phonological skill.

## 2. Methods

### (a) Subjects

Two hundred and thirty-eight students (99 male; mean age 11.1 years, s.d. 0.3 years) from the St Thomas More Catholic School, Gateshead, UK, were tested. The school is non-selective and admits pupils from a local catchment area. The cohort was selected solely on the basis of belonging to year 7. The work aimed at auditory processing in typical language development, and data from 28 students were excluded owing to a discrepancy between their language skill and full-scale intelligence quotient (FSIQ), the basis for the DSM-IV (Diagnostic and Statistical Manual of Mental Disorders) criterion for dyslexia: 16 had reading and spelling scores that were lower than their FSIQ by 15 or more standard points and 12 had a reading or spelling score plus an associated measure (non-word reading, reverse digit span) with such a discrepancy. The analyses presented are based on the remaining 210, with the only reason for missing data points being occasional absence or the failure to complete a test.

### (b) Auditory testing

Auditory testing was performed in a quiet classroom environment, one class at a time (*n* = 16–30) on separate computers with external soundcards and headphones. Four pitch perception tasks, four rhythm and timing tasks and four tests of timbre perception based on modulation (figure 1; see the electronic supplementary material, Methods) were performed in three sessions of 60–75 min each; testing time was 5–15 min per task. The task design was as similar as possible across tasks. All tasks used a two-alternative forced-choice paradigm and most of them an adaptive procedure, except the ‘same–different’ pitch tasks with fixed difficulties. Adaptive tests estimated the 70.9 per cent correct threshold for the tested parameter; ‘same–different’ tests the sensitivity index *d*' (see the electronic supplementary material, Methods).

#### (i) Pitch

The first three pitch tasks used 250 ms pure tones (figure 1*a*), the fourth used synthetic piano melodies. The *basic pitch change detection task* required the subject to indicate which of two pairs of pure tones included a change in frequency. The *local and global change detection tasks* (40 trials each, same–different) required the subject to indicate whether two four-tone sequences were ‘the same or different' [41]. In the local task, the change in frequency of one note preserved the pattern of ‘ups and downs'. In the global version, a change in pattern was created along with the change in frequency. The *key violation detection task* from the Montreal Battery for the Evaluation of Amusia [54] required the subjects to indicate whether two melodies were ‘the same or different’, with the change in one note violating the key structure. The first three tasks test the perception of pitch changes found in either speech or music, while the fourth is specific to the tonal structure of Western music.

#### (ii) Rhythm

All four rhythm and timing tasks [55] used 500 Hz 100 ms pure tones (figure 1*b*). The *basic, single-interval task* required subjects to indicate which of two tone pairs comprised the ‘longer gap’. In the *isochrony-deviation detection task*, subjects were required to indicate which of two otherwise isochronous five-tone sequences contained a lengthening or ‘extra gap’. In the *regularity detection task*, subjects were required to indicate which of two nine-tone sequences was ‘overall more regular’. In the *metrical pattern discrimination,* subjects were required to decide which of three rhythmic sequences was ‘different, or wrong’ owing to a distortion in its metrical rhythm. Inter-onset intervals ranged from 180 to 660 ms, corresponding to time intervals between stress events in speech [12,56,57] and musical beats [58,59].

#### (iii) Timbre

The four tasks of timbre perception included two FM detection tasks, implicated in reading ability previously [14,17], plus dynamic modulation (DM) detection and discrimination tasks based on spectral–temporal modulation relevant to speech [1,11] (figure 1*c*). In the *FM detection tasks*, subjects were required to identify a tone modulated at a rate of 2 Hz, sounding ‘ringing or wobbly’, or 40 Hz, sounding ‘rough’ against a ‘flat-sounding’ unmodulated 500 Hz reference. In the *DM detection and discrimination task*, subjects identified an ‘alien or laser-like’ modulated sound against an unmodulated noise reference, and one with a higher versus a lower modulation rate, respectively. Stimulus duration was 1000 ms in the FM and 500 ms in the DM tasks.

### (c) Neuropsychological testing

Phonological and intellectual abilities were tested one-to-one over a 1 h period. The combination of six standard tests of phonological skill included: written rhyme decision (PALPA), spelling (WIAT), word reading (WIAT), non-word reading (WIAT), non-word repetition (WMTB-C) and backward digit span (WMT-C). FSIQ was assessed based on two subtests of verbal and non-verbal IQ each (WASI). (For test details, see the electronic supplementary material.)

### (d) Data analysis

Statistical analysis used rank-ordered data and is described in detail in the electronic supplementary material, Methods. In summary, separate principal component analyses (PCAs) were carried out on the auditory and phonological data, and correlations assessed between the first, general auditory and phonological principal components (PCs), and between the task-specific auditory measures and phonological skill.

## 3. Results

The aims of this work were to demonstrate the general relationship between auditory and phonological skill and the specific role for sequence analysis. We describe below the separate PCAs for the auditory and phonological data, the general correlation between the first auditory and phonological PCs that explained most of the variance within either dataset and the task-specific correlations between single auditory measures of auditory sequence analysis and phonological skill. The detailed raw data are presented in the electronic supplementary material, tables S1 and S2 and figures S1 and S2.

### (a) Principal component analysis

**Figure 2.**
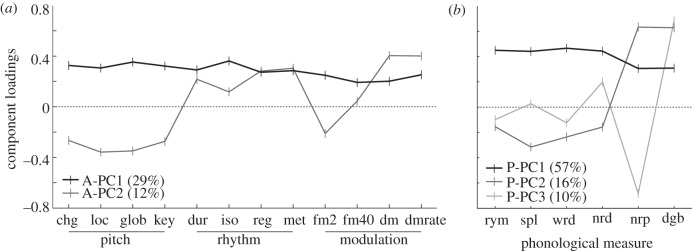
Principal component (PC) loadings. Displayed are the loadings for those components of either dataset that explained at least 10% of the variance of the data from all those individuals who completed all 12 auditory (*n* = 172) and six phonological tasks (*n* = 176), respectively. (*a*) Auditory (A-PCn). (*b*) Phonological (P-PCn). Task abbreviations: pitch: chg, basic change detection; loc, glob, local and global change detection; key, key violation. Rhythm: dur, single time-interval duration discriminations; iso, isochrony-deviation detection; reg, regularity detection; met, metrical pattern discrimination. Modulation: fm2 and fm40, 2 and 40 Hz FM detection; dm, DM detection; DMs, dmrate discrimination; rym, rhyme decision; spl, spelling; wrd, word reading; nrd, non-word reading; nrp, non-word repetition; dgb, digit span backward.

The auditory PCA was conducted across the three domains of pitch, time and timbre, including the data from all individuals that completed all 12 tasks (*n* = 176). The first PC explained 29 per cent of the variance with almost uniform loadings across tasks (A-PC1; figure 2*a*, black line) and will be referred to as the general auditory component. The second component (A-PC2; figure 2*a*, grey line) explained 12 per cent of the variance, with high loadings on the rhythm and timing and DM tasks.

The phonological PCA was performed on the data from those individuals that completed all six standard tests of phonological skill (*n* = 172). These tests can be assumed to draw on underlying mechanisms, such as phonological representations, phonological store and phonological working memory [60–62]. The first PC explained 57 per cent of the variance, with almost uniform loading across all six tasks (P-PC1; figure 2*b*, black line), and will be referred to as the general phonological component. The second and third PCs (P-PC2 and P-PC3; figure 2*b*, dark and light grey lines) explained 16.0 per cent and 10.0 per cent, respectively. P-PC2 had high loadings specifically for non-word repetition and digit span backwards, and may reflect the phonological store. P-PC3 had a negative loading for non-word repetition and a positive one for digit span backwards, possibly reflecting phonological working memory.

### (b) Correlation between auditory and language skill

**Figure 3.**
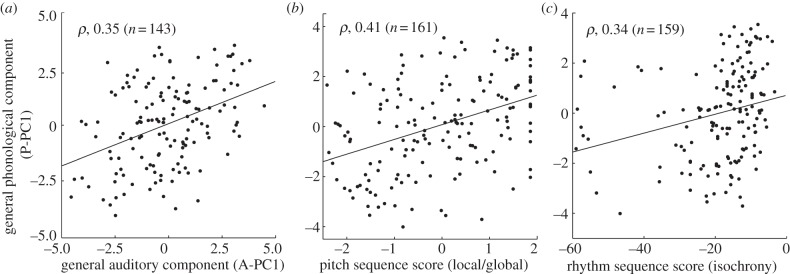
Correlations and the first phonological PC (P-PC1) and (*a*) auditory PC1 (A-PC1); (*b*) the combined pitch score for local and global short-sequence tasks (from PCA on the two measures); (*c*) the rhythm score (threshold in per cent inter-onset interval for deviation detection in short isochronous sequences).

A significant correlation was demonstrated between the general auditory and the general phonological component (A-PC1 and P-PC1), with a *ρ* of 0.35 (*p* < 0.001) before and 0.22 after (*p* < 0.01) controlling for non-verbal IQ, explaining over 12 per cent and nearly 5 per cent of the variance, respectively (figure 3*a*). No correlation was found between the first auditory and the second or third phonological component, or the second auditory and any phonological component.

In order to test which auditory abilities are most relevant, correlations were sought between the task-specific auditory measures and the general phonological component (P-PC1). Significant correlations explaining at least 5 per cent of the variance independently of non-verbal IQ, and surviving Bonferroni correction, were found exclusively for tasks of short-sequence analysis. No such correlations were found for any of the basic or more complex or musically oriented tasks. Within the pitch domain, correlations with P-PC1 were found for the local change-in-sequence task, with a *ρ* of 0.39 before and 0.27 after controlling for non-verbal IQ (both, *p* < 0.001), and for the global change-in-sequence task, with a *ρ* of 0.35 (*p* < 0.001) and 0.23 (*p* < 0.01), respectively (both, *n* = 161). As performance on the two pitch tasks was strongly correlated (*ρ*, 0.59, *p* < 0.001, *n* = 195), the relationship with phonological skill might be owing to a common mechanism of pitch-sequence processing. To assess such a mechanism, we conducted another PCA only on the two pitch measures and extracted the first PC as a combined score explaining 80 per cent of the variance. This score also correlated significantly with P-PC1, with a Spearman's *ρ* of 0.41 before and 0.28 after controlling for non-verbal IQ (both, *p* < 0.001; *n* = 161; figure 3*b*). It may be noteworthy that both pitch-sequence tasks were subject to a slight ceiling effect, as was the combined score. While a significant correlation was demonstrated, the ceiling effect might obscure a true correlation stronger than that observed. Within the rhythm and timing domain, a significant correlation with P-PC1 was found specifically for the isochrony-deviation detection in short sequences, with a *ρ* of 0.34 before and 0.32 after controlling for non-verbal IQ, respectively (both, *p* < 0.001; *n* = 159; figure 3*c*). Importantly, both the combined pitch score and the rhythm score correlated significantly with the P-PC1 also after correcting for the other measure and non-verbal IQ, with a residual *ρ* of 0.19 (*p* < 0.01) and 0.26 (*p* < 0.001) (*n* = 150), respectively.

Correlations between auditory and phonological task-specific measures were also tested (see the electronic supplementary material, tables S3 and S4), with the main finding of patchy, yet significant correlations that occurred almost exclusively for those auditory tasks that correlated with P-PC1: local and global change-in-sequence pitch tasks, and isochrony-deviation detection. In addition, the metrical rhythm task correlated with rhyme decision, while regularity detection showed a trend towards correlation with a few phonological measures.

To summarize, a significant general relationship was shown between auditory and phonological language skill between first PCs (that explained 29% and 57% of the variance, respectively) where both components showed high loadings on all tasks of either dataset. A specific role was shown for auditory measures of short-sequence analysis in pitch and time, but not for the more basic or the more complex measures as tested here. In order to exclude possible confounding effects of cognitive, educational or social factors, we conducted an analysis of post-code-based deprivation indices and found a correlation with phonological measures and IQ but virtually no effect on the auditory measures and their relationship with phonological skill (see the electronic supplementary material, Results).

## 4. Discussion

### (a) The relationship between auditory and language skill

This work demonstrates a significant, limited relationship between auditory and phonological language skill in early adolescence. The correlation between the general components extracted from the phonological and the auditory data was of moderate-to-low effect size, explaining 12 per cent of the variance with inclusion of the effect of non-verbal intelligence, and only 4 per cent when this was excluded. This small residual correlation between the general phonological component (accounting for nearly 60% within the phonological data) and the auditory one (accounting for about 30% within the auditory data) suggests a weak but significant link. There are a number of interpretations of this. The auditory capacity extracted in our PCA might correspond to a general auditory perceptual or attentional mechanism relevant to the performance of all of the auditory tasks, equally so for the domains of pitch, time and timbre and for the analysis over different time scales, from single sounds to complex sequences. If that is the case, this general auditory mechanism has a limited but significant relationship with phonological skill. Another possibility that must be considered is that the first component extracted from the auditory data may reflect a cognitive skill that is not specifically auditory, and that this aspect of cognition is also relevant to phonological skill.

In the analysis based on individual tasks, a specific relationship was demonstrated: robust correlations with phonological skill were found exclusively for auditory tasks of short-sequence processing. After partialling out non-verbal intelligence, the correlation between the general phonological component and short-sequence tasks in pitch and time explained 7 per cent and 11 per cent of the variance, respectively. Unique residual variance explained by sequence analysis in pitch and time after accounting for shared variance was 4 per cent (pitch: *ρ*, 0.19; *p* < 0.01) and 6 per cent (rhythm: *ρ*, 0.26; *p* < 0.001), respectively. No such correlation was shown between phonological skill and the perception of single sounds or longer, more complex or musical sequences. The findings support the idea that the link between auditory and phonological skill is limited and depends on the level of ‘acoustic complexity’ considered [25]. The level of acoustic complexity relevant to phonological skill may further vary with age and between different cohorts, and it will be of considerable interest to carry out further studies to investigate these factors.

The importance of auditory processing to language skill has been controversial over 30 years. The inconsistency between reports is concerning [63] given that some tasks are now used in training programmes with limited success [31–33,64–66]. Part of the ongoing controversy may result from the tendency to focus on specific aspects of auditory processing in small and select samples [10,14,17,22,23,30,41–43,45,67]. Two recent studies in larger, non-selected samples suggested very limited roles for basic auditory processing [27,28] and emphasize temporal tasks typically based on two sounds [28]. The present study is a large comprehensive assessment of single-sound versus sound-sequence analysis, a level rarely studied before.

### (b) A specific role for auditory sequence analysis

The specific correlation between short-sequence analysis in pitch and time and phonological skill may reflect their relevance in the ‘parsing’ of the speech stream [4,38,39]. Certain developmental theories of language acquisition are based on ‘suprasegmental’ units rather than individual phonemes [68]. Difficulties with phonological awareness and literacy in dyslexia have consequently been argued to be owing to ‘a perceptual deficit in the mechanisms used to extract the suprasegmental attributes of the speech stream’ [42]. The correlation demonstrated here supports our hypothesis that the sequence tasks used here tap into mechanisms of suprasegmental analysis critical to typical language development.

In the pitch domain, previous reports describe correlations between phonological skill and a combination of local and global change detection in 4–5-year olds [40], and global pitch-contour processing in young adults [41]. The current study in early adolescents shows a correlation for both global and local change detection. The presence of correlation for the local task might reflect greater power and greater range of ability in the present cohort. Other methodological differences were the use of simpler, four-tone sequences here compared with six-tone sequences used by Foxton *et al*. [41], and the fact that the transposed version of the global task (arguably a ‘purer’ global task) was too difficult for the present cohort. Alternatively, the difference in correlations may reflect developmental differences in the relationship: a change in the levels of auditory analysis relevant to phonological analysis during childhood and adolescence. This remains to be explored further.

In the timing and rhythm domain, we demonstrated a robust correlation with phonological skill for the processing of short, isochronous rhythms of roving tempo. This ability has not, to our knowledge, been looked at before, and the results support a critical role for it in normal language development. While no previous work has assessed such generic analysis of short rhythmic sequences, a correlation with rhythmic amplitude-modulation processing has been shown in dyslexic and typically developing children [42,45]. The previous work assessed the ability to process the shape of individual events comprising a sequence rather than the timing between events. The ability to detect rhythmic deviations between events as tested here may aid the extraction of important cues in the suprasegmental time structure of the incoming speech stream and the parsing into smaller units [39,69]. The detection of a roughly regular beat in longer sequences, however, only showed a trend to correlation with phonological skill. This might be owing to the overall difficulty that the students had with this task or alternatively, reflect a lack of relevance of this ability in typical English language development at this age. It will be of interest to further assess this ability in different age groups and in native speakers of other languages, in particular those with more regular, syllable-timed structure [56,70]. Supporting evidence for the importance of rhythmic analysis at such longer sequence levels is provided by recent work on differences in evoked potentials for speech envelope in poor compared with normal readers [71].

The lack of correlation between phonological skill and the more musical tasks was not surprising. Both the key violation and metrical rhythm tasks assess the analysis of features that are specific to the structure of Western music but not present in speech. The perception of key violation requires some knowledge of the Western scale in the pitch dimension and was tested in a way that does not depend critically on the processing of pitch contour over time. The metrical task used an adaptive-tracking paradigm based on one particular rhythm presented repeatedly at different tempi, in order to test metrical-beat processing to its limit. Language does not have a metrical structure like music [72], and would not require such precise temporal analysis. Previous work in dyslexic and typically developing children has demonstrated a correlation between language ability and metrical rhythm discrimination for once-presented sequences with a fixed difficulty level [43]. The absence of robust correlations for our metrical discrimination task could be explained by its adaptive design measuring metrical-beat-based timing to its limit of precision in a way that is not relevant to the tested phonological language skills. Overall, our tests do not directly support a close link between phonological skill and the processing of musical stimuli. The correlations we observe emerge at a more generic level of pitch and timing analysis that may be equally relevant to speech and music [44,73,74].

### (c) The limited role for basic auditory processing

The perception of pitch, time, FM or DM requires the analysis of fundamental acoustic features present in the speech signal. The absence of correlation between these basic auditory measures and phonological skill was unexpected in comparison to previous studies reporting significant effects for pitch discrimination [13] and FM detection [14–17], but such correlations are a matter of debate. A re-analysis of the subsamples of typical and dyslexic adults from one of the previous studies did not show a relationship [17,63]. Two other reports evaluating FM detection deficits in adult dyslexics suggest that those may not be causal or necessary [16,63]. Further studies failed to replicate a consistent relationship in FM detection in children at high risk of dyslexia [23], or backward-masking in teenagers or children with dyslexia or SLI [10,24,25]. Neither single-interval timing nor DM perception has been assessed in relationship to phonological language skill before. They have been argued to be important aspects in the speech signal [1,11,12,57,75], but it is debatable how critical the analysis of all of the tested basic features is to phonological processing. For example, sinusoidal 40 Hz FM has been argued to correspond to fast changes at the phonemic level of formant transitions. However, FM of a pure tone is quite different from the shift in a formant, an energy maximum in a harmonic sound with multiple frequency components, and given that average phoneme transitions occur over 30 ms, a lower rate might be more relevant [76,77]. In an effort to match speech properties in our stimuli, we included dynamic spectro-temporal modulations (DM) with wider peaks more like formants, using parameters corresponding to those in speech [1,11]. However, there was also no correlation with phonological skill for the processing of these stimuli. One possible explanation is that highly accurate analysis of basis parameters is not needed for adequate speech analysis and phonological processing, an interpretation supported by other recent studies in large cohorts [27,28]. Arguments have in fact been made for an essential role of feature extraction and categorization, rather than auditory-processing accuracy [35,78,79]. One other explanation for the present lack of correlation might relate to the prolonged testing of a large number of subjects in a classroom environment, where despite close supervision an overall lack of attention cannot be excluded. One session lasted up to 75 min and typically included four tasks, interspersed with short breaks and group-level instructions. During task performance, groups of five students were supervised by one researcher to ensure participants' compliance and concentration. This cannot prevent attentional effects however, and the relevance of attention to our data cannot be dismissed. This aspect merits further investigation, but there are currently few reliable and specific measures of auditory attention [27,80–82].

## 5. Conclusion

Our findings suggest a specific relevance of short-sequence analysis to language skill in early-adolescents. This may reflect the relevance of auditory sequence analysis to the parsing of the speech stream. Further research in typically developing subjects of different ages is required. The work suggests a level of auditory processing that may also be relevant to abnormalities of language development: should that prove to be the case, a new type of auditory training for language disorders is suggested based on pitch and rhythmic sequences.
